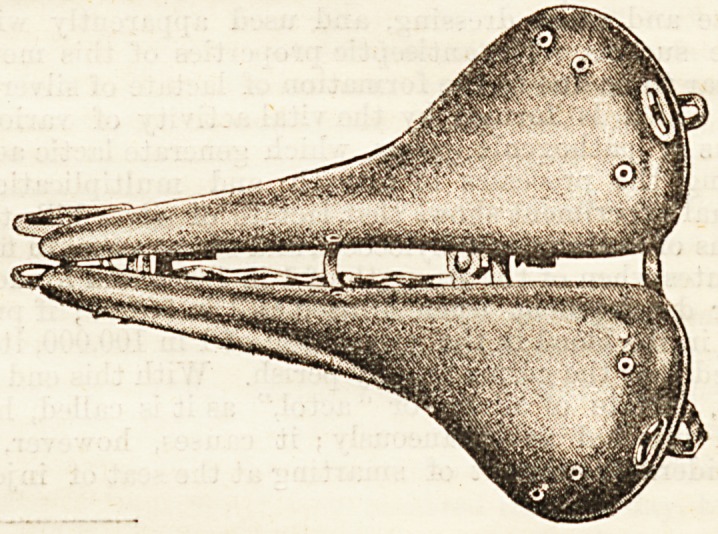# New Appliances and Things Medical

**Published:** 1896-10-03

**Authors:** 


### NEW APPLIANCES AND THINGS MEDICAL.

[We shall be glad to receive, at our Office, 28 & 29, Southampton Street, Strand, London, W.C., from the manufacturers, specimens of all new preparations and appliances which may be brought out from time to time.]

### "OXINE."—A CONCENTRATED INVALID SOUP.

(Leslies, Limited, Bond Court House, Walbrook, London, E.C.)

The above firm has forwarded us a specimen of this new concentrated invalid soup. It is put up in the form of rectangular tabloids of sufficient size to make half-a-pint of soup, and for the preparation all that is necessary is to cut it up into five or six small pieces and dissolve in the necessary amount of water, and boil for five minutes with a small quantity of salt. The result of this simple operation is the possession of a really excellent clear soup, containing a large proportion of the fragrant and nutritious constituents of the meat from which it is prepared. From trials to which we have submitted this new preparation, it appears to be well tolerated by the most delicate and irritable stomachs, and further than this, it certainly possesses nutritive and stimulating properties of a high degree. Although for domestic use the tablet form is exactly what is required, for hospitals and institutions the manufacturers inform us that it may be procured in bulk at 10s. per lb.

### NEW OINTMENT INTRODUCER.

(Reynolds and Branson, 13, Briggate, Leeds.)

This little instrument, of which we give an illustration, is the invention of Mr. Nevill, of Leeds, and his designs have been put into execution by Messrs. Reynolds and Branson. It appears to possess many useful innovations on the older forms. The cylinder is of large size, holding about an ounce of ointment; it possesses a uterine tube and a nozzle that will fit an ordinary catheter. It can be readily taken to pieces and cleaned; it can be used for rectal purposes by the patient himself without fear of damage; it is easy to fill, and contains a number of doses, which can be injected by a specially graduated piston-rod and armature.

### FAC-SIMILE HUMAN MILK.

(Welford and Sons, Elgin Avenue, Maida Vale, W.)

The above firm, whose methods and resources for supplying hygienically pure milk are known to all residents in London, have more recently turned their attention to the preparation of a milk for infants and children, which, as far as chemical analysis can show, is almost identical with the human variety, the form and character of the coagulum of the casein is practically the only particular in which it has not physiological identity. So-called "humanised milk" is often made by diluting the product of the cow with water, adding a little cream, and perhaps a little sugar. The method adopted by Messrs. Welford is different. Their first care is to get the best milk, and by the special feeding of cows at their own farms at Willesden and Harlesden, they have the means of doing so under their own control. Having once obtained a milk rich in cream and other solid constituents, the most important step in the process is completed. The subsequent standardisation to the equivalents of human milk is simply a matter of care and attention. The name given by the firm for this standard milk for infants is "Fac-simile Human Milk"—a name which in our opinion it very fairly merits. Messrs. Welford also undertake to supply peptonised, sterilised, asses and goats' milk for those infants who are unable to assimilate cow's milk in its simple or humanised form.

### UNIVERSAL ADJUSTABLE SPLINT.

(Messrs. Allen and Hanbury, London.)

This splint is intended to take a place in the splint cupboard, and thus be ready for immediate use in place of the plaster or other methods of obtaining rigidity and rest for injured limbs which are resorted to when the ordinary ready-made splints are not suitable. It can be readily understood that an adjustable splint would be of great servi e in field service and in country practice. The splint is in the form of the concave metal plates resting on rods, on which the screws are fixed which govern the adjustment. Each plate is capable of a complete rotary movement separate from the other, so that the support can be fixed with the pressure applied at any part of the upper or lower limb which is desired, whilst the bend of the joint is provided for at the joint of the splint, which works by means of a cogwheel, and permits of adjustment to the various angles allowed by the action of the wheel, and the limitation of this movement can be quickly ascertained before use by the surgeon. The splint can be altered in position when in use without removing it from the limb. It is not unduly heavy, being made of aluminium, and can be easily cleaned, as the parts are readily separated. The idea is novel, and has much to recommend it, where more tedious processes of obtaining a malleable splint are inconvenient.

### MALTED FOOD FOR INVALIDS AND INFANTS.

(The British Malt Product Company, 98, Bermondsey Street, E.C.)

This new preparation of the above company has been submitted to us for trial and report. Owing to the artificial digestive processes to which the food is submitted, the original starch is completely malted, and reduced to a condition in which it can be readily assimilated by the digestive tract. We have tried it in the case of an infant four weeks old, which was unable to digest or retain any form of cow's milk, diluted, humanised, condensed, or predigested—it was readily taken, and apparently had good flesh-forming qualities. As is well known by those who have had experience in infant feeding, there is no golden road to success. One infant will thrive on a food which may be poison to another. It will therefore be found of value to those who are interested in this important branch of practice to give this new preparation a trial until milk can again be digested. For invalids, especially such as require an agreeable and fattening food, the new preparation has also its value, and being to a certain extent a complete food for adults, it may safely be relied upon for a time to maintain the animal function single-handed, when other foods are rejected.

### THE PATTISON CYCLE SADDLE.

(Pattison Cycle Saddle Syndicate, 15, Copthall Avenue, E.C.)

Whilst there are very few medical men who do not advocate cycling in moderation, the use of the ordinary saddle has admittedly certain injurious tendencies. The Pattison cycle saddle thoroughly obviates these objections, without affecting speed or balance. Our illustration gives the general form, and it will be seen that the saddle is completely divided in the centre, towards which, being concave, the surface inclines. The weight is not only thrown on the ischial bones, as it should be, but the saddle is also adjustable to the comfort of each rider by means of a screw, which widens or diminishes the opening, thus securing a wide or narrow seat at will. The peak of the saddle is placed upon a spring, which prevents undue vibration and jolting, and relieves the forward pressure known to be so injurious. The grip is in no way interfered with. The saddle is handsome, light, and durable, being made of the best materials, whilst great care has been exercised to secure good workmanship. We think that for these reasons the Pattison saddle will be generally recommended by medical men. The showrooms are conveniently situated at 90, Shaftesbury Avenue.

## Figures and Tables

**Figure f1:**
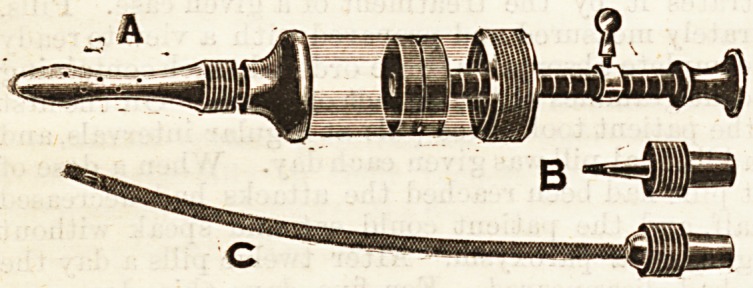


**Figure f2:**